# Integration Analysis of Transcriptome and Proteome Reveal the Mechanisms of Goat Wool Bending

**DOI:** 10.3389/fcell.2022.836913

**Published:** 2022-04-01

**Authors:** Yue Liu, Yangyang Ding, Zhanfa Liu, Qian Chen, Xiaobo Li, Xianglan Xue, Yabin Pu, Yuehui Ma, Qianjun Zhao

**Affiliations:** ^1^ Key Laboratory of Animal (Poultry) Genetics Breeding and Reproduction, Ministry of Agriculture and Rural Affffairs, Institute of Animal Sciences, Chinese Academy of Agricultural Sciences (CAAS), Beijing, China; ^2^ The Ningxia Hui Autonomous Region Breeding Ground of Zhongwei Goat, Zhongwei, China; ^3^ Department of Animal Breeding and Reproduction, College of Animal Science and Technology, Yunnan Agricultural University, Kunming, China; ^4^ CAAS-ILRI Joint Laboratory on Livestock and Forage Genetic Resources, Institute of Animal Sciences, Chinese Academy of Agricultural Sciences (CAAS), Beijing, China

**Keywords:** zhongwei goat, skin, proteomics, transcriptomics, PRM, wool bending

## Abstract

Zhongwei goat is a unique Chinese native goat breed for excellent lamb fur. The pattern of flower spikes of the lamb fur was significantly reduced due to the reduction of the bending of the hair strands with growth. In order to explore the molecular mechanism underlying hair bending with growth, we performed the comprehensive analysis of transcriptome and proteome of skins from 45-days, 108-days and 365-days goat based on TMT-based quantitative proteomics and RNA-seq methods. In the three comparison groups, 356, 592 and 282 differentially expressed proteins (DEPs) were screened, respectively. KEGG pathway analysis indicated that DEPs were significantly enriched in a set of signaling pathways related to wool growth and bending, such as ECM-receptor interaction, PI3K-Akt signaling pathway, PPAR signaling pathway, protein digestion and absorption, and metabolic pathways. In addition, 20 DEPs abundance of goat skin at three development stages were examined by PRM method, which validated the reliability of proteomic data. Among them, KRT and collagen alpha family may play an important role in the development of goat hair follicle and wool bending. COL6A1, COL6A2, CRNN, TNC and LOC102178129 were identified as candidate genes based on combined analysis of transcriptome and proteome data and PRM quantification. Our results identify the differential expressed proteins as well as pathways related to the wool bending of Zhongwei goats and provide a theoretical basis for further revealing the molecular mechanism underlying wool bending of goats.

## Introduction

Goats provide considerable materials for the animal fibre industry. Wool has natural crimped fibers, and the curvature is one of the important index for evaluating the wool quality. The degree of curvature is critical to judge the grade of wool between different breeds or individuals, which can be used as one of the characteristics of breed protection and breeding. Zhongwei goat is a Chinese native breed renowned for excellent lamb fur, which has been put into the Chinese livestock and poultry genetics resources protection list. The hair strands of the Zhongwei goat lamb fur are curved since birth. Nevertheless, the wool-bending accompanied with natural and exquisite patterns disappear gradually within two or 3 months, which result in dramatic decrease of economically value of goat fur (Li H and Feng, 2015). The mechanisms underlying hair-curly phenotype change of Zhongwei goat in a short-term was still little known (Xiao et al., 2019).

Proteomics is a powerful tool to reveal the composition, location, changes and interactions of proteins in cells, tissues or organisms (Pandey and Mann, 2000), the research includes protein and functional patterns. Furthermore quantitative proteomics proves far more challenging compared with nucleotides sequencing. Proteins display a wide range of variation in abundance, making low-abundant proteins easily masked by high-abundant proteins with similar mass or chemical properties (Smith et al., 2019). Comparative proteomics methods based on mass spectrometry, such as Label-free, iTRAQ, SWATH, PRM, allow us to identify proteins with significantly changed expression levels under specific conditions on a large scale (Bourmaud et al., 2016; Li et al., 2020). Joint analysis of proteomics and other omics provide more useful information for exploring the mechanisms of wool bending and hair follicle development.

In recent years, increasing studies have provided insights into wool growth of sheep and goats as well as other species have been reported (Plowman et al., 2020). For instance, the difference in protein abundance was explored and Keratin-related proteins (KRT), related to hair growth and fatty acid synthesis (FAB) were identified to be potentially related to changes in fiber characteristics based on iTRAQ-based proteomics analysis of 18 samples of sheep and goats (Li et al., 2018). Several gene families, including fibroblast growth factor (*FGF*), *WNTs*, insulin-like growth factor (*IGF*) were found to be associated with wool growth through the analysis of gene expression microarray and proteomic data of the body-side skin (more wool growth) and groin skin (no wool growth) of Chinese Aohan fine wool sheep (Zhao et al., 2017). Three secreted proteins including apolipoprotein-A1, galectin-1 and lumican have been identified to be enriched in embryonic skin which is essential to induce new hair follicles (Fan et al., 2018). Several studies suggest that the wool curl mechanism is consistent with human hair bending (Harland, 2018). Previous study showed hair curvature was caused by the distributions of different cell types (Medland et al., 2009; Harland et al., 2018). It has been discovered that the Trichohyalin gene (*TCHH*) affect European hair bending (Wu et al., 2016). Several genes were determined to be related to curly hair phenotype, such as *LPAR6* (Manakhov et al., 2020), *CAP1* (Liu et al., 2017) and *KRT* (Liu et al., 2018; Salmela et al., 2019). However, the mechanisms of Zhongwei goat wool-curly phenotype change with growth are still little known.

The present study is aimed to determine the changes of proteins and mRNAs of goat skin at different developmental stages and reveal the mechanism underlying wool bending of Zhongwei goat. To this end, we performed comparative analysis of the transcriptome and proteome of Zhongwei goat skin at three time points (D45, D108, D365) with divergent wool bending pattern based on tandem mass tag (TMT) quantitative proteomics and RNA-seq approaches. We identified a set of proteins, genes as well as signalings pathways involved in wool bending. This study provides useful transcriptomic and proteomic expression dataset and contribute to understanding the mechanism of wool bending in Zhongwei goat.

## Results

### Identification of Proteins in Goat Skin

**FIGURE 1:**
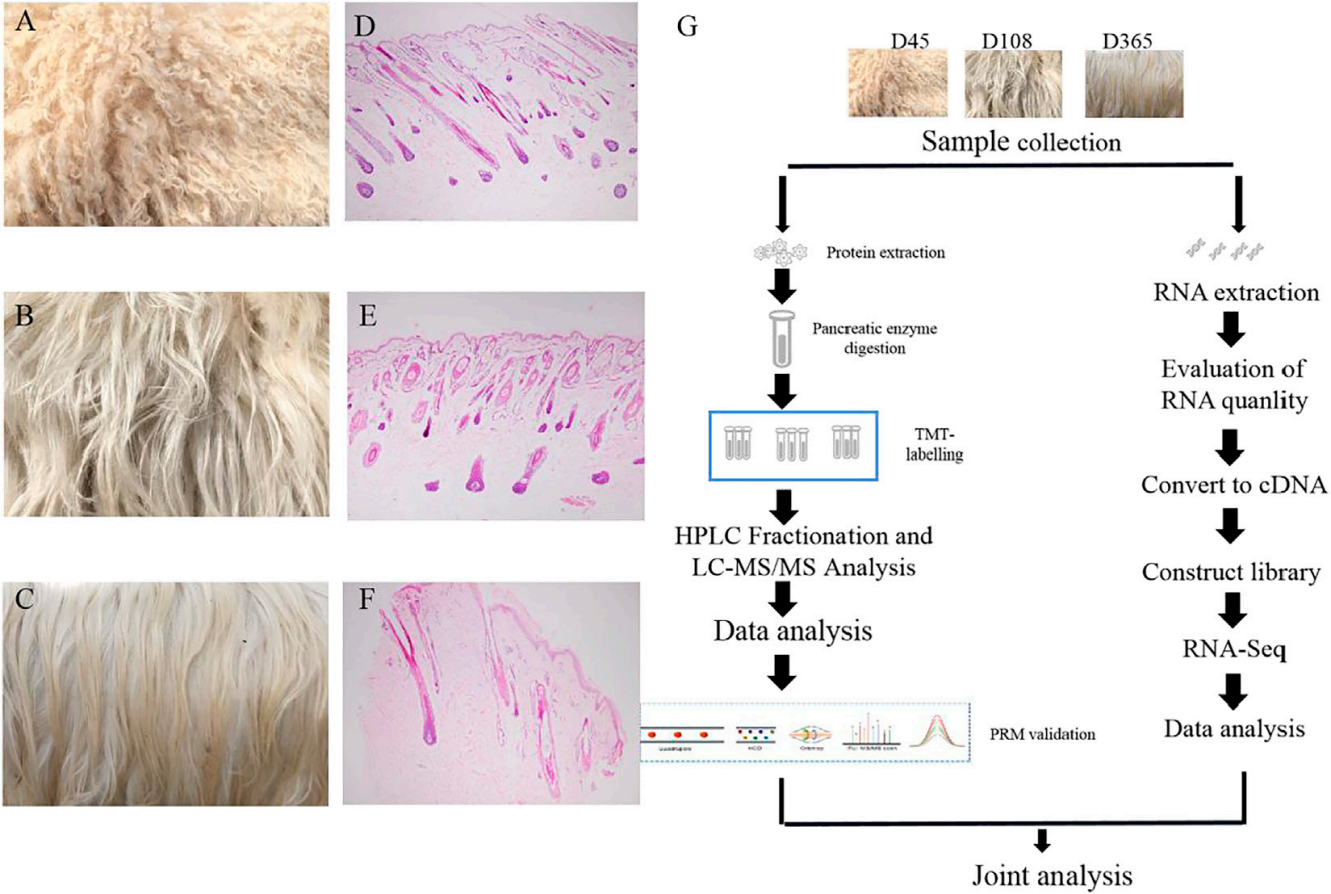
Wool phenotype of Zhongwei goat and workflow **(A)** Zhongwei goat wool at day 45. **(B)** Zhongwei goat wool at day108. **(C)** Zhongwei goat wool at day 365. **(D)** 45-days-old goat hair follicle. **(E)** 108-days-old goat hair follicle. **(F)** 365-days-old goat hair follicle. **(G)** workflow of quantitative proteome and transcriptome analysis of skin using TMT LC-MS/MS and RNA-seq methods.

To investigate protein dynamic changes during goat wool growth, we performed Zhongwei goat skin proteomic analysis in three periods using TMT-based quantitative method. The proteome and transcriptome profiling of Zhongwei goat lamb fur with highly curved hair (D45, T1), slightly curved hair (D108, T2) and straight hair (D365, T3) were constructed (Figures 1A–G). Totally, 33,965 peptides and 31,553 specific peptides were identified. Among three groups, a total of 4,994 proteins were identified, and 3798 were quantifiable (Supplementary Table S1).

**FIGURE 2:**
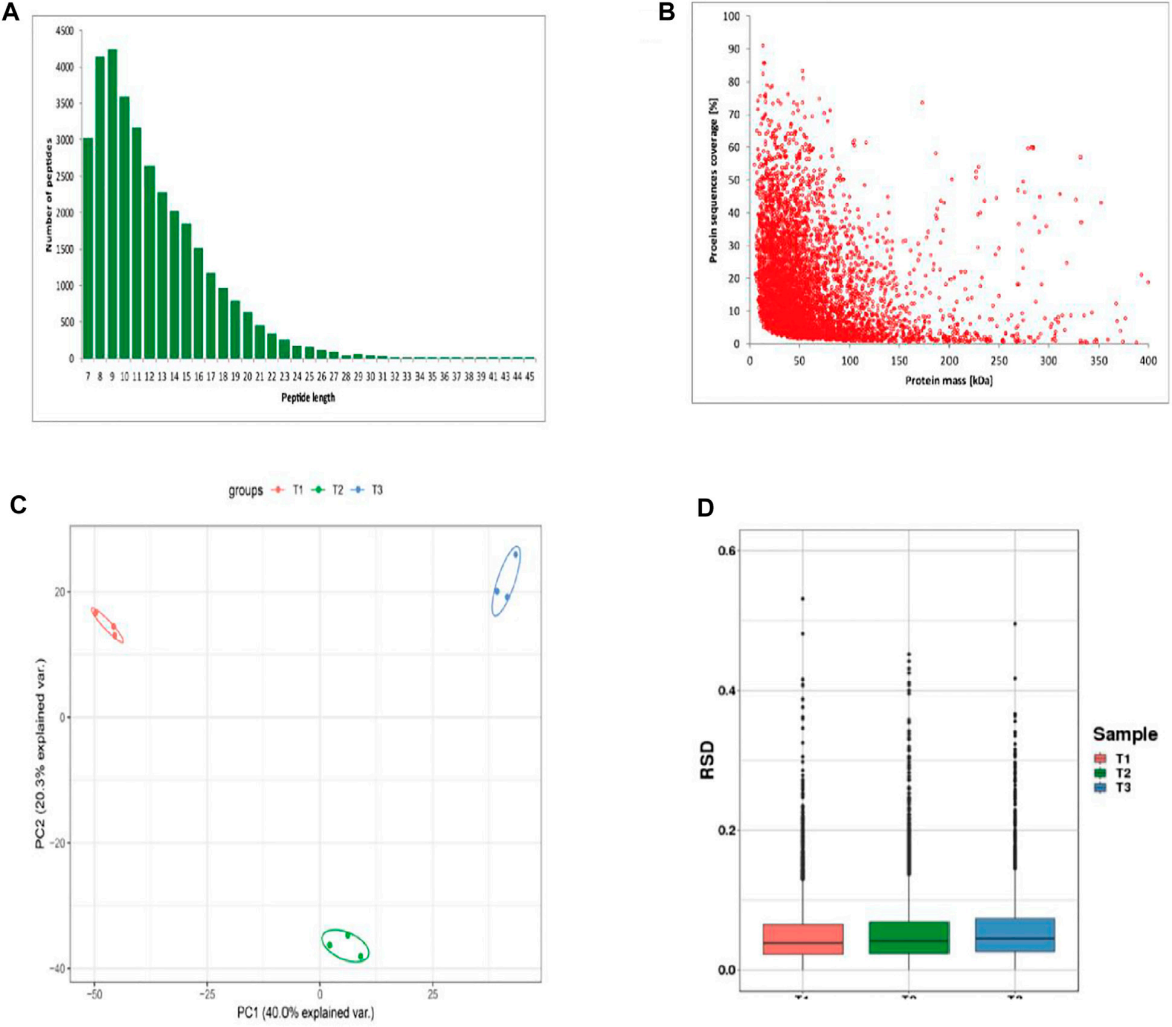
Identification of proteins of skins from Zhongwei goats **(A)** Peptide length distribution. **(B)** protein sequence coverage distribution. **(C)** Two-dimensional scatter plot of quantitative principal component analysis of protein among samples. **(D)** Box plot of quantitative RSD distribution of protein among samples.

The length distribution of peptides showed most peptides ranged from 7 to 12 amino acids (Figure 2A). The counts of identified peptides showed a decreasing from 0 to 400 kDa (Figure 2B). To assess the quantitative consistency of the protoemics data, we clustered the protein based on principal component analysis and relative standard deviation approaches. The PCA result showed the three biological replicates in the each three periods were clustered together, and the protein quantification varies greatly among samples at the three periods (Figure 2C). The box plot of the relative standard deviation (RSD) of quantitative protein values between replicate samples, demonstrated the high reproducibility of our datas (Figure 2D).

### Differential Protein Screening

**FIGURE 3:**
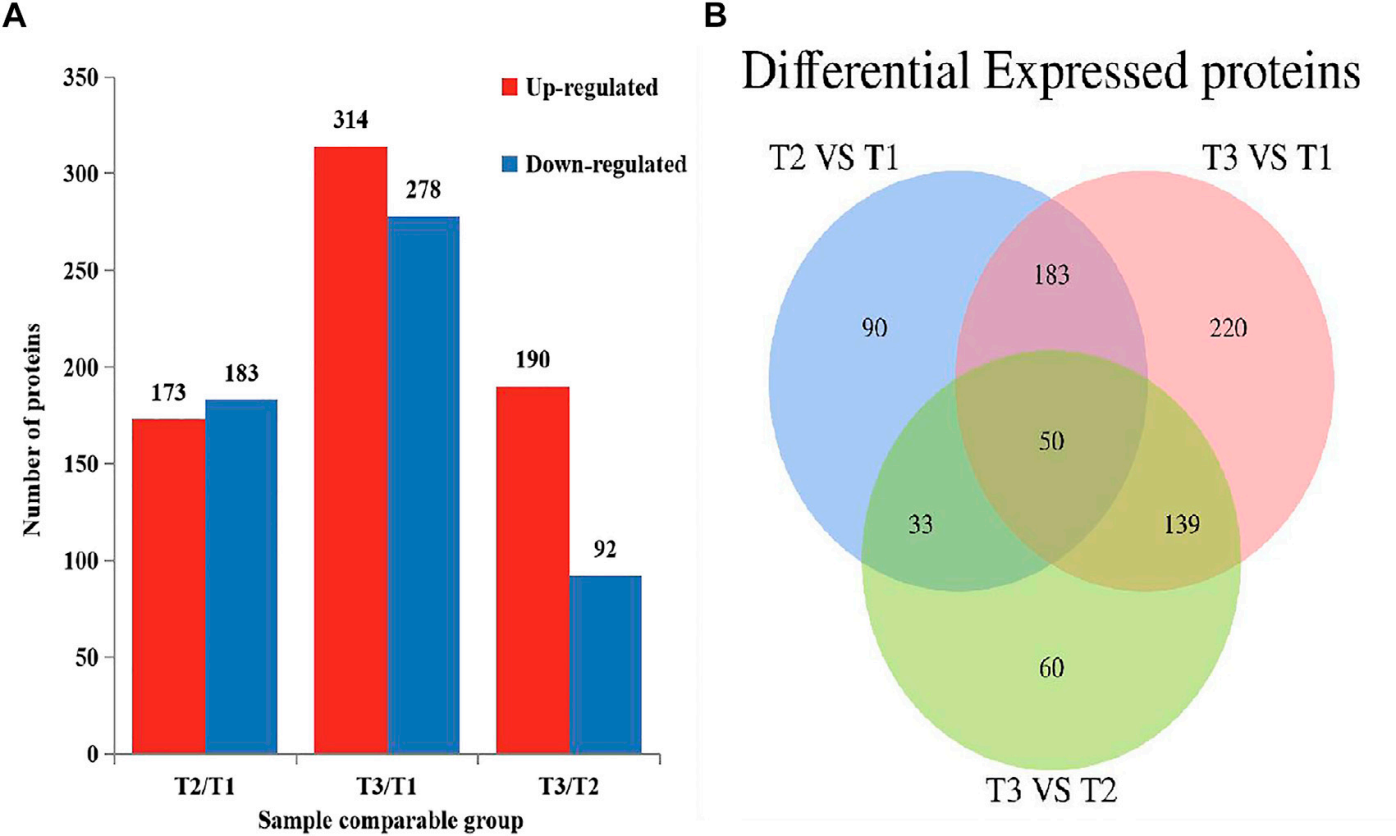
Differential expressed proteins in three comparison groups (*p* < 0.05, |fold change|>1.3). **(A)** The number of DEPs. **(B)** Venn diagram of DEPs in Zhongwei goat skin at three developmental stages.

To explore the protein difference of goat skin with various wool-bending type, we determined the differentially expressed proteins (DEPs) among three groups. *p* < 0.05 and |fold change|>1.3 was used as the screening condition for DEPs. A total of 356 (T2 VS. T1), 592 (T3 VS. T1) and 282 (T3 VS. T2) DEPs were identified among three groups (Figure 3A), and 50 overlapped DEPs were observed in the three comparison groups (Figure 3B). The most abundant differential proteins were observed between samples from D45 (T1) and D365 (T3). Among them, 278 proteins in skins of group T1 had higher expression than that in group T3, and 314 proteins in skin of group T1 had lower expression levels compared with that in T3 (Supplementary Figure S1A–C).

### Functional Enrichment Analysis of Differentially Expressed Proteins

**FIGURE 4:**
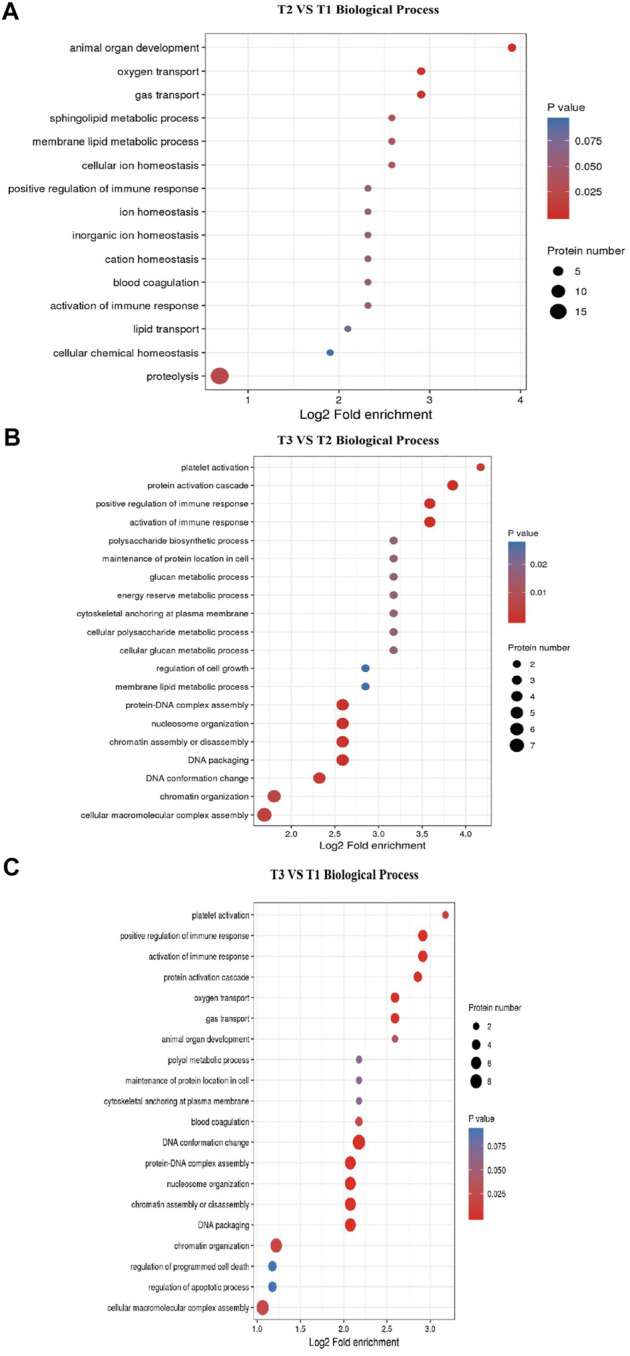
GO terms enrichment for Biological Process. **(A)** T2 VS. T1 Biological Process items. **(B)** T3 VS. T2 Biological Process items. **(C)** T3 VS. T1 Biological Process items.

GO and KEGG enrichment analysis of DEPs were performed to explore the potencial function of DEPs identified in this study. The GO enrichment result showed that all categories were enriched and the biological process items were mainly enriched (Supplementary Figure S2). The top 5 GO terms for biological process in T2 VS. T1 comparison group included animal organ development (GO:0048513), sphingolipid metabolic process (GO:0006665), oxygen transport (GO:0015671), gas transport (GO:0015669) and membrane lipid metabolic process (GO:0006643). Other GO terms for biological processes were related to metabolism, such as proteolysis (DNPEP, CTSZ, NLN, NRDC, etc.). In T3 VS. T2 comparison group, activation of immune response (GO:0002253), chromatin assembly or disassembly (GO:0006333), protein-DNA complex assembly (GO:0065004), nucleosome organization (GO:0034728) and DNA packaging (GO:0006323) were enriched. Specifically, some GO terms related to transcription were significantly enriched, such as cellular macromolecular complex assembly and chromatin organization (Figure 4). Meanwhile, as shown in Supplementary Figure S3, most of the DEPs were located in extracellular region (GO:0005576) and cytoskeletal part (GO:0044430) cellular component terms. These DEPs maintain the cellular shape and play an important role in hair follicle development. These results suggested that hair follicle development and wool growth was accompanied by protein formation and hydrolysis.

**FIGURE 5:**
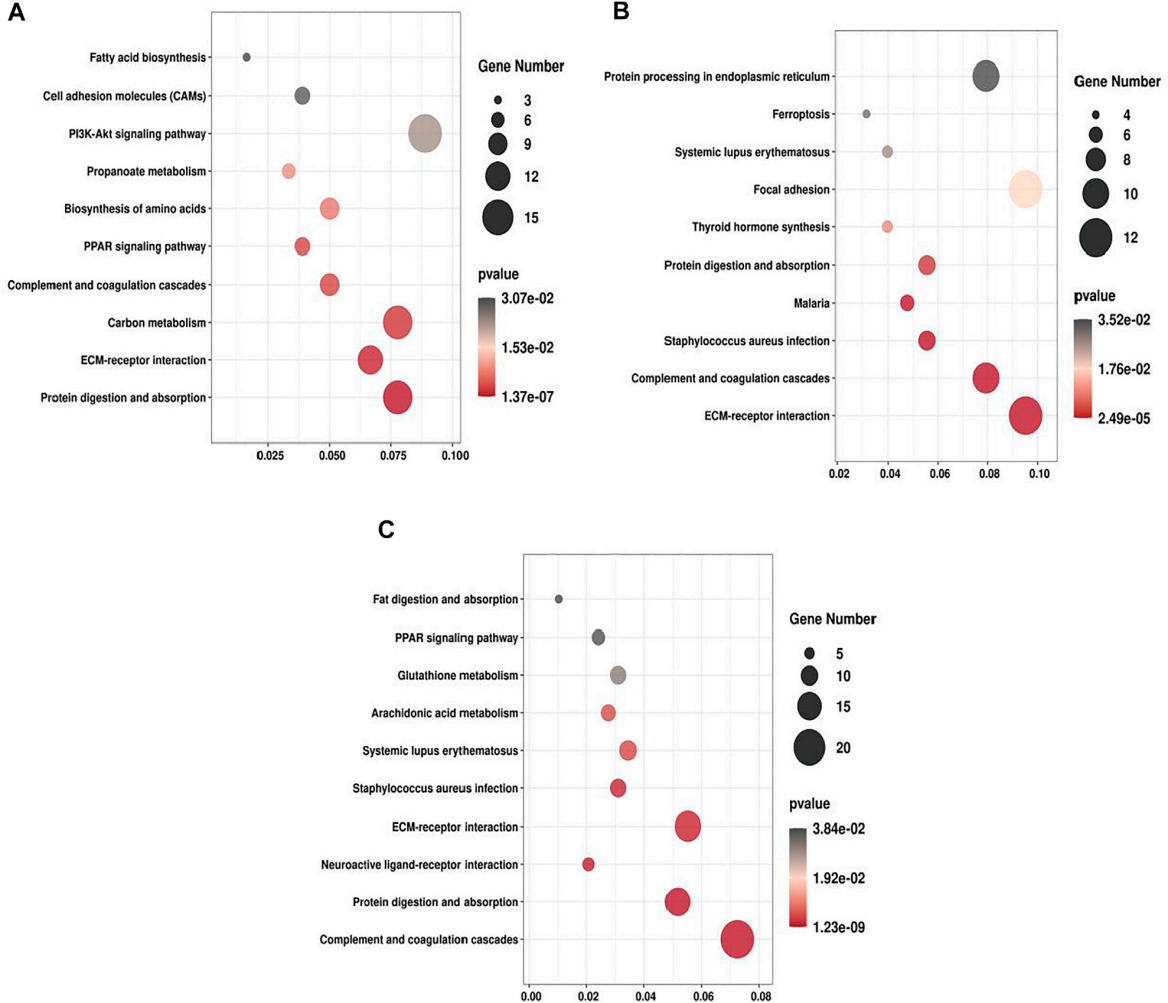
KEGG pathway enrichment of DEPs. **(A)** T2 VS. T1. **(B)** T3 VS. T2. **(C)** T3 VS. T1.

We performed KEGG enrichment analysis for all the DEPs and the up- and down-regulated DEPs, respectively. In the three groups, the DEPs were mainly enriched in ECM-receptor interaction, Protein digestion and absorption and PPAR signaling pathway (Figure 5). For T2 VS. T1 group, up-regulated DEPs were significantly enriched in ECM-receptor interaction and PI3K-Akt signaling pathway, whereas down-regulated DEPs were mainly enriched in PPAR signaling pathway and Fatty acid degradation. For T3 VS. T2 group, upregulated DEPs were significantly enriched in Protein processing in endoplasmic reticulum and Thyrold hormone synthesis. Majority of downregulated DEPs were enriched in Relaxin signaling pathway and Fluid shear stress and atherosclerosis (Supplementary Figure S4). As ECM-receptor interaction, PI3K-Akt signaling pathway and protein digestion were mostly significantly enriched in all three groups, thus these pathways may exert vital roles in Zhongwei goat hair growth and bending.

### Differential Protein Network Interaction in Related Signaling Pathways

**FIGURE 6:**
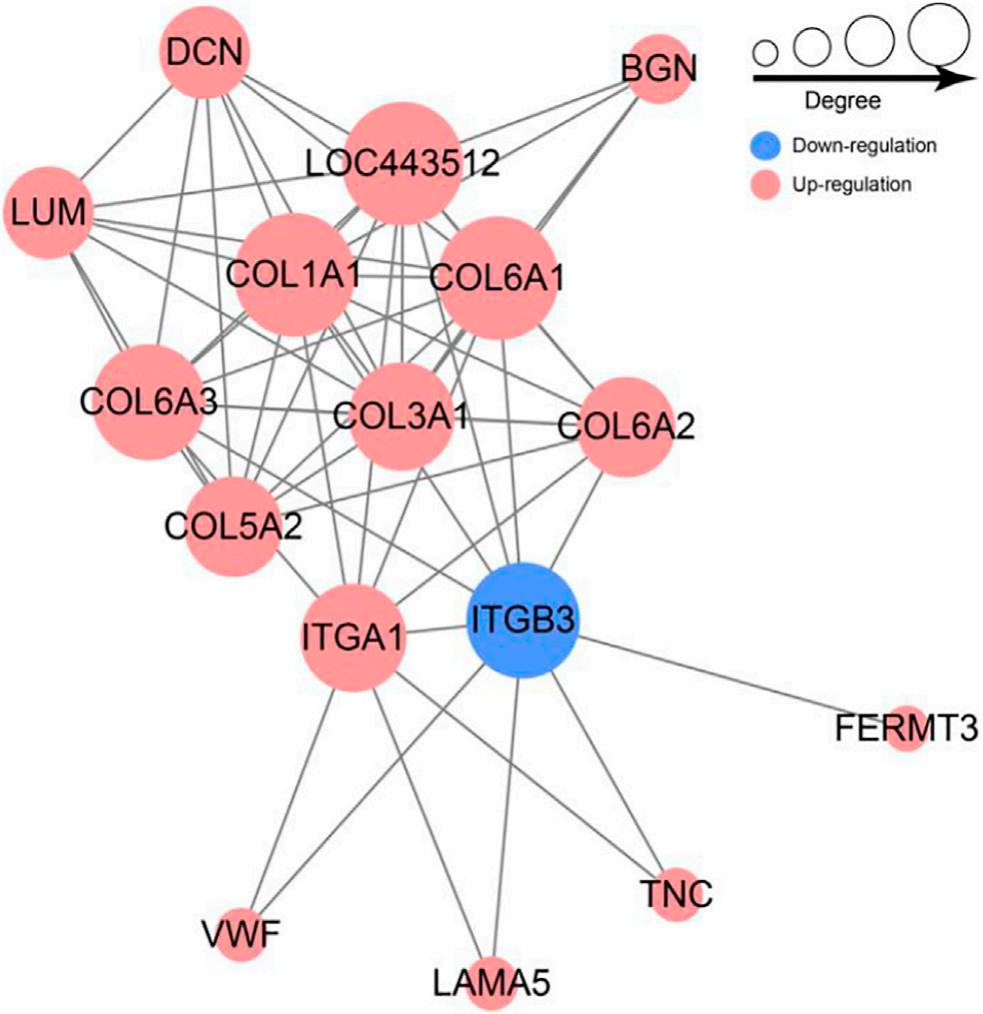
Network of differentially expressed protein involved in ECM-receptor interaction and PI3K-Akt signal pathway.

To further investigate the associations among these DEPs, a Protein-protein interaction (PPI) network was constructed using the STRING database. The PPI network of DEPs contains 56 edges and 16 nodes (Figure 6). Collagen family protein (COL1A1, COL3A1, COL5A2, COL6A2, COL6A1, COL6A3), collagen binding protein (ITGA1) and signal protein (ITGB3, BGN) interacted strongly with each other. In addition, we found these proteins were significantly enriched in ECM-receptor interaction and PI3K-Akt pathway based on PPI and KEGG enrichment results (Figure 6). Furthermore, majority of proteins were up-regulated with growth. Collectively, we speculated that the ECM-receptor interaction pathway and PI3K-Akt pathway might be involved in regulating goat wool growth and bending.

### PRM Quantitative Results

According to the expression level and the function of the DEPs, 20 candidate proteins were selected for PRM quantification to verify the accuracy of the proteomic data in three comparison groups, of which 18 proteins were quantified. For instance, compared with group T1, the expression level of COL1A1 was dramatically higher in other groups. Conversely, KRT72, a member of Keratin protein family, known as the major component of wool fiber, was highly expressed in group T1. The alternation of these DEPs expression may be associated with wool bending phenotype. The overall trend of the PRM detection ratio results and TMT data analysis ratio results was consistent, indicating that the proteomic data were repeatable and reliable (Table 1).

**TABLE 1:** PRM result compared with TMT-based quantitative result.

Protein accession	Gene name	T2/T1 ratio (PRM)	T2/T1 *p*-value	T2/T1 ratio (TMT)	T3/T1 ratio (PRM)	T3/T1 *p*-value	T3/T1 ratio (TMT)	T3/T2 ratio (PRM)	T3/T2 *p*-value	T3/T2 ratio (TMT)
XP_013827532.2	LOC102176161	0.74	4.38E-03	0.5	0.59	4.38E-03	0.25	0.8	6.11E-02	0.49
XP_017901608.1	CRNN	0.34	5.06E-04	0.58	0.58	2.77E-03	0.75	1.71	7.51E-04	1.3
XP_017920804.1	MRC2	0.58	2.26E-02	0.76	1.63	8.83E-03	1.38	2.83	1.66E-04	1.82
XP_005684195.1	FBP1	1.34	6.60E-03	1.41	6.5	1.52E-06	3.73	4.86	1.05E-06	2.64
XP_005681373.2	CFI	1.43	1.15E-03	1.44	1.82	3.62E-05	1.9	1.27	1.07E-03	1.32
XP_005701908.2	KRT74	0.62	3.53E-02	0.78	0.63	3.57E-02	0.67	1.02	8.24E-01	0.85
XP_017921364.1	LOC108638461	3.22	2.44E-05	1.6	7.59	5.81E-06	3.1	2.36	4.12E-05	1.94
XP_017908170.1	TNC	1.65	1.79E-04	1.64	2.84	7.43E-06	2.26	1.71	6.16E-05	1.38
XP_017920382.1	COL1A1	2	9.24E-04	1.73	2.72	7.51E-04	2.42	1.36	1.03E-02	1.4
XP_017896254.1	LOC102178129	1.75	3.74E-05	1.52	2.79	3.82E-05	2.08	1.6	4.30E-04	1.37
XP_017903218.1	KRT80	1.28	6.76E-03	1.17	0.8	5.46E-03	0.87	0.63	9.01E-04	0.75
XP_013832621.2	KRT72	0.57	1.55E-02	0.71	0.45	5.19E-03	0.63	0.8	1.47E-01	0.89
XP_005692313.1	LOC102191166	0.24	2.36E-05	0.39	0.37	6.03E-05	0.56	1.57	1.17E-03	1.44
XP_005681801.1	ALB	0.51	1.71E-05	0.76	1.29	2.97E-04	1.39	2.52	1.25E-06	1.83
XP_005675518.2	CP	0.75	4.01E-03	0.76	1.49	1.19E-03	1.46	1.97	2.24E-04	1.91
XP_017921952.1	LOC102186111	1.51	5.01E-02	1.33	4.64	1.11E-03	1.88	3.08	2.15E-03	1.42
XP_017907820.1	COL6A2	1.46	5.35E-03	1.38	2.75	4.08E-05	2.24	1.89	4.71E-05	1.63
XP_017907809.1	COL6A1	1.42	2.09E-02	1.48	2.55	7.12E-05	2.23	1.8	7.47E-04	1.51

### Transcriptomic Analysis

**FIGURE 7:**
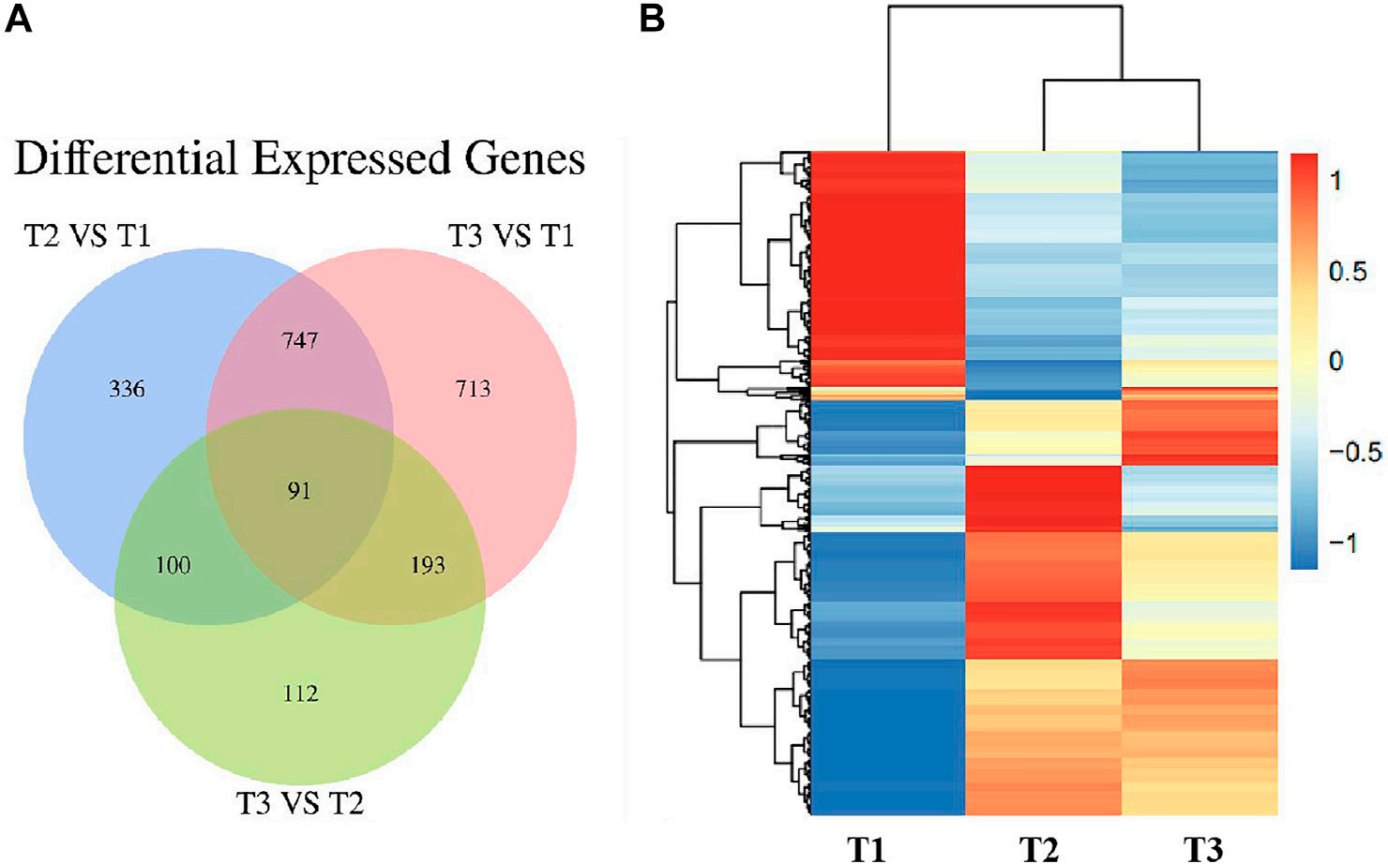
**(A)** Venn diagram of differentially expressed genes in Zhongwei goat skin at three developmental stages. **(B)** Cluster analysis of gene expression in three groups.

To further validate the protein expression abundance patterns in goat skin, RNA-seq was conducted to compare the transcriptome in Zhongwei goat skin tissues with different wool bending characteristics. Nine RNA libraries for skin samples were constructed and raw paired-end reads were generated using the Illumina Hiseq2000 sequencer. Totally, 22,115 genes were identified in the skins of Zhongwei goats, of which, 1,274, 1744 and 496 differential expression genes (DEGs) were determined in comparison of T2 VS. T1, T3 VS. T1, and T3 VS. T2 (q ≤ 0.05, |FC|>1.5). In addition, 91 overlapped DEGs were observed in the three comparison groups (Figure 7A). Cluster analysis of DEGs showed that most of DEGs at D45 were downregulated, and the gene expression trends at D108 and D365 were relatively similar (Figure 7B).

### Integrated Analysis of Transcriptome and Proteome

**FIGURE 8:**
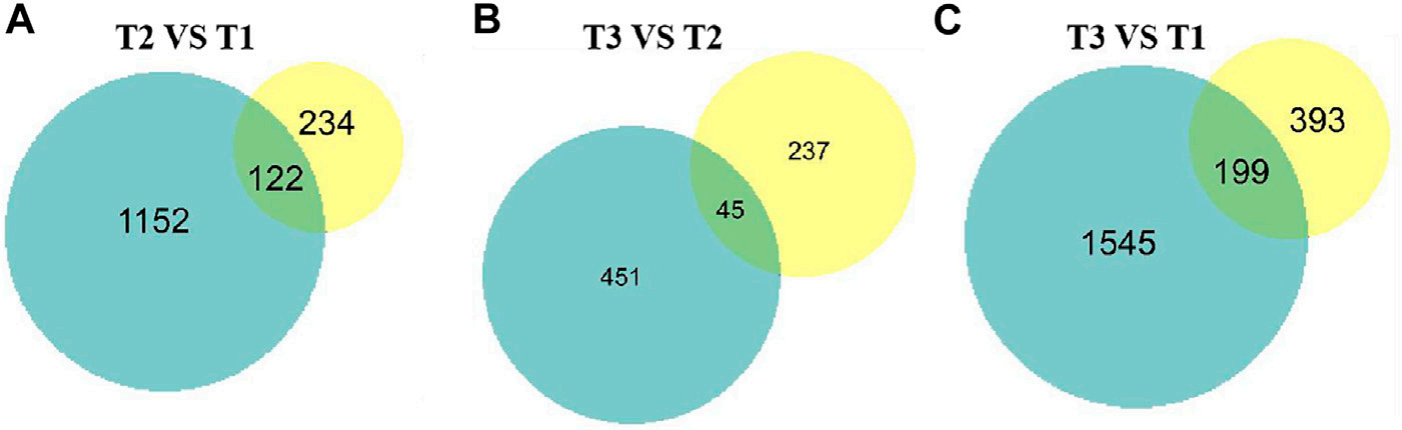
Proteome and transcriptome expression Venn diagrams of three different sets of wool during curving period. Blue pie represent transcriptome, yellow pie represent proteome. **(A)** T2 VS. T1. **(B)** T3 VS. T2. **(C)** T3 VS. T1.

To intersect the correlation of transcriptome and proteome data, the DEPs and DEGs were compared in three groups. Of 1,219 DEGs and 356 DEPs, a total of 122, 199, 45 over lapped genes were identified among three groups (Figure 8). Furthermore, we analyzed the change trend of all DEGs at both transcriptomic and proteomic level. Based on mRNA and protein expression level, we found a total of 96 genes including 69 downregulated and 27 upregulated genes were in a same trend. 34 DEGs were upregulated in the transcriptome but downregulated in proteome, while 36 DEGs were decreased at the transcriptomic level but increased at the proteomic level.

**FIGURE 9:**
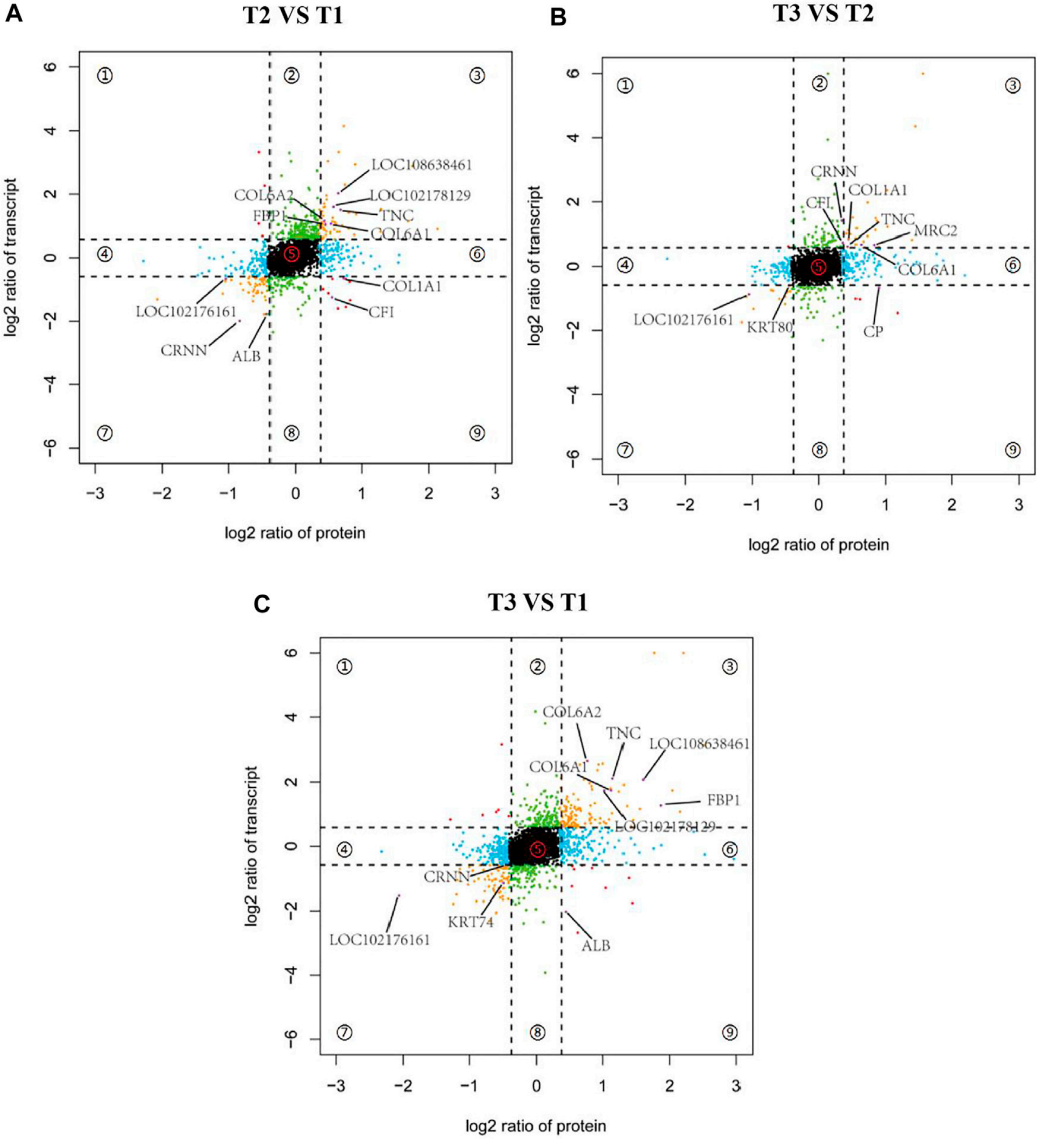
Nine-quadrant diagram of differentially expressed genes between transcriptome and proteome. Quadrants 1 and 9 indicate that the expression trends of the two omics are opposite. Quadrants 2 and 8 indicate genes that only express differences in the transcriptome. Quadrants 3 and 7 indicate that the expression trends of the two omics are consistent. Quadrants 4 and 6 Indicates that only differentially expressed proteins are expressed in the proteome, and quadrant 5 indicates genes that are not expressed in both omics. **(A)** T2 VS. T1. **(B)** T3 VS. T2. **(C)** T3 VS. T1.

To further screen potential candidate genes involved in goat wool bending, nine-quadrantal diagrams were drawn based on transcriptome and proteome results, and then annotated the nine-quadrant results combined with the PRM data. In the three groups, over 12% genes and proteins showed a differential expression patterns. A small number of proteins showed lower abundances than the related RNAs in first, second and forth quadrants (Quadrants 1, 2 and 4). Proteins and RNAs enriched in the sixth, eighth and ninth quadrants showed higher abundances than the related RNA in the sixth quadrant, which might be caused by post-transcriptional or translation-level regulation (Quadrants 6, 8 and 9). Combined with the PRM quantification results, we found that the annotated proteins were mostly located in the quadrants 3 and 7, and that these genes were consistent with the corresponding protein expression pattern, implying that these proteins were transcriptionally regulated (Figure 9). T2 VS. T1 group included a total of eleven overlapped genes. nine genes had the same expression trend. A total of ten overlapped genes were observed in the T3 VS. T1 group, nine of which had the same expression trend. T3 VS. T2 group had nine overlapped genes, eight of which had the same expression pattern (Figure 9). COL6A1 and TNC reflected the same expression trend in the three groups, which implied COL6A1 and TNC protein levels are transcriptionally regulated.

### Candidate Gene Verification

**FIGURE 10:**
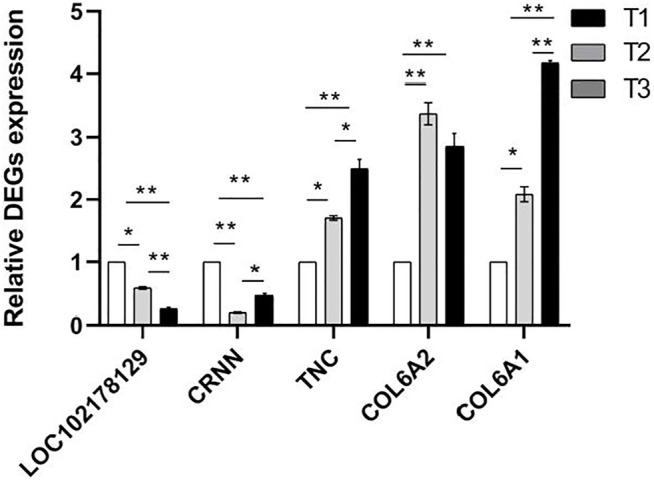
Relative mRNA expression levels of proteins using qPCR method. NOTE: * means *p* < 0.05, ** means *p* < 0.01.

In view of the predicted functions, enriched biological processes, fold changes, PPI network of these DEPs, five mRNAs (LOC102178129, CRNN, TNC, COL6A2, COL6A1) expression level were examined in the skin tissue at three developmental stages using qPCR method (Figure 10). The results were agreement with transcriptomic and proteomic data (Supplementary Figure S5). It showed that TNC, COL6A2 and COL6A1 were up-regulated, while CRNN had the lowest expression in the skin of D108 and the highest expression in the skin of D45.

### Effect of Interfering COL6A1 on DPCs Proliferation

**FIGURE 11:**
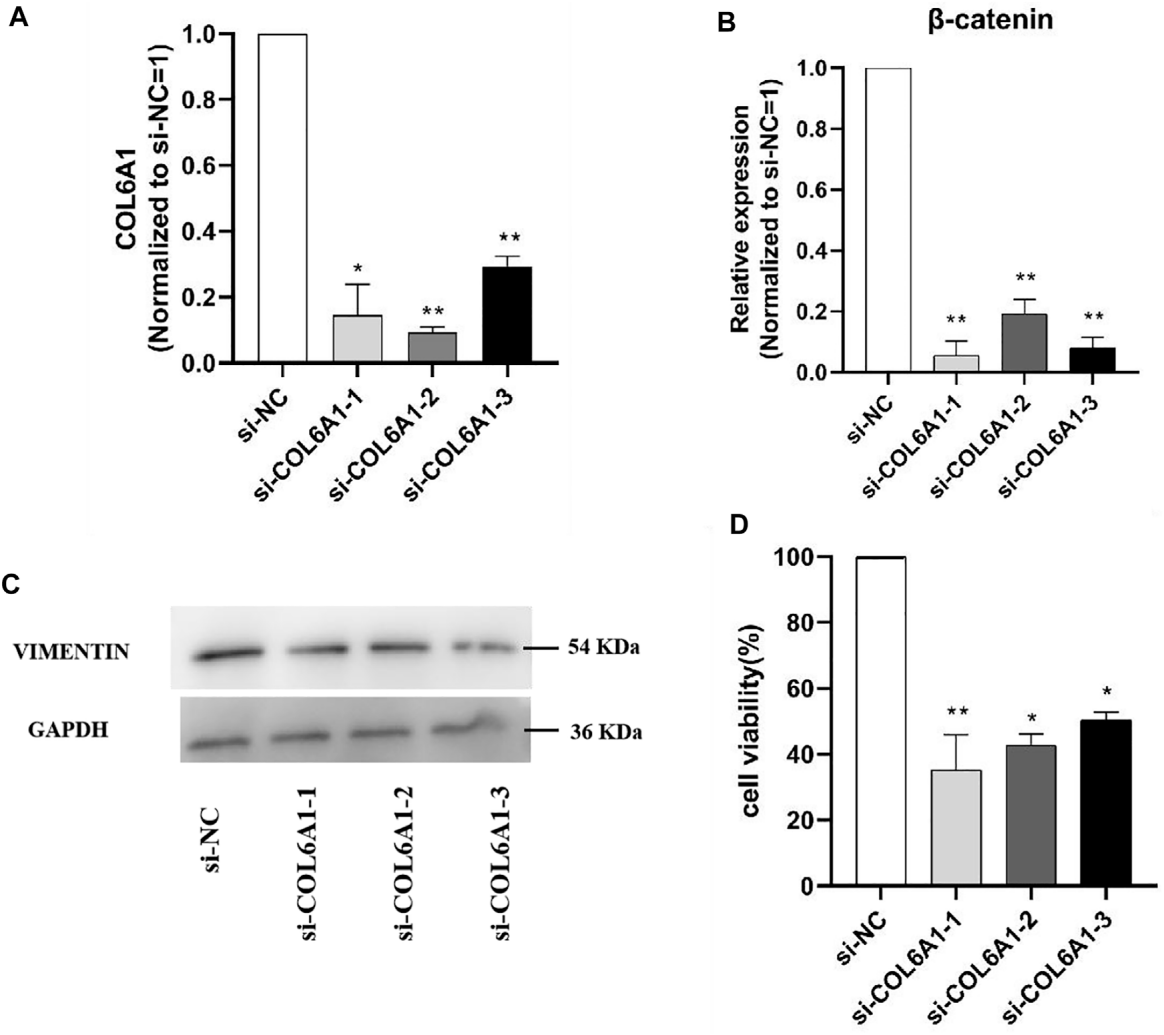
Effect of interfering COL6A1 on DPCs proliferation. **(A)** qPCR results of COL6A1 in DPCs at 48 h when COL6A1 was inhibited using siRNA. **(B)** qPCR results of cell proliferation after siRNA transfection. **(C)** Western blots of COL6A1 knockdown DPCs at 48 h. **(D)** Cell proliferation was assessed after 48 h treatment using MTT assay. NOTE: * means *p* < 0.05, ** means *p* < 0.01.

To determine the effect of COL6A1 on dermal papilla cells (DPCs), we transfected the scrambled sequence (siRNA) and the negative scrambled sequence (si-NC) with DPCs. The mRNA level of β-catenin and protein abundance of VIMENTIN were significantly decreased upon COL6A1 depletion (Figure 11A,B,C), and β-catenin and VIMENTIN highly expressed in skin, which play important roles in DPCs development. Furthermore, MTT analysis of DPCs proliferation was performed to investigate the effect of COL6A1. The result showed that COL6A1 knockdown decreased cell proliferation (Figure 11D). Therefore, we speculated that COL6A1 effect wool growth or wool phenotype through regulation of hair follicle growth cycle.

## Discussion

The curvature of hair affects wool quality and economic value, thus it is very necessary to reveal the mechanism of wool bending. Wool bending is determined by hair follicle growth and development which is regulated by a serious of genes and pathways. Previous studies have uncovered several genes and proteins involved in follicles development and wool growth of sheep (Zhao et al., 2017; He et al., 2020). However, studies on the proteomic changes and the molecular mechanism underlying goat wool bending have rarely been performed. To understanding the mechanism of wool bending, we combined a series of cutting-edge technologies such as TMT labeling, high-performance liquid chromatography fractionation technology, and mass spectrometry-based quantitative proteomics technology to explore the proteins and mRNA profiling dynamics of Zhongwei goat skin with different curving-degrees in three developmental periods.

In the current study, TMT-based quantitative proteomics revealed that 4,994 proteins were obtained in all samples, and 50 proteins were denoted as candidate genes for wool growth based on differential analysis. In addition, the DEPs were associated with keratin filament, peptidase regulator activity, animal organ development, and membrane lipid metabolic process, which has been reported in other species like human (Lai et al., 2021) and goat (Gao et al., 2016). Based on KEGG pathway enrichment some pathways related to wool growth and hair follicle development were identified, such as Protein digestion and absorption, Fatty acid biosynthesis, ECM-receptor interaction and PI3K-Akt signaling pathway. Notably, the ECM-receptor interaction and PI3K-Akt signaling pathway were significantly enriched in all the three comparison groups. Previous studies have reported that the ECM-receptor interaction signaling pathway modulate hair follicle development through regulating cell proliferation and migration, which affected wool growth (Zhang et al., 2020a; Haage et al., 2020). Therefore, we speculate ECM-receptor interaction and PI3K-Akt signaling pathways play a major role in goat wool bending.

Previous has been reported that abnormal activity of pathways and polymorphisms in regulatory molecules lead to follicle diversity and hair curvature. In addition, research have shown that the effect of hair bending was more easily explained by differential growth rates at opposing sides across follicle (Vandenberg and Levin, 2009; Harland et al., 2018). Recent years, PI3K-Akt (Nie et al., 2018), Wnt (Zhang et al., 2020b), EDA/EDAR (Wu et al., 2020), EGFR/TGF-α (Cheng et al., 2010), BMP (Bai et al., 2021), EDA/NF-κB (Tomann et al., 2016), Shh (Shwartz et al., 2020) and other classic signaling pathways have been found to directly or indirectly related to wool bending (Ding et al., 2020). Research also found that ECM-receptor interaction and PI3K-Akt pathway were related to hair follicle development (Wang et al., 2021). In our study, the ECM-receptor interaction and PI3K-Akt pathway were significantly enriched, especially the ECM-receptor interaction pathway mainly enriched in three groups. PI3K-Akt signaling pathway was also found to be associated with Tan Sheep curly wool characteristics (Chen et al., 2020). Several proteins were included in the ECM-receptor interaction pathway such as collagen, laminin and fibronectin, furthermore, those gene differential expressed between primary and secondary hair follicle dermal papilla cells lead to dermal papilla enlargement (Zhu et al., 2013). The collagen, an important member of fibrin family, is the most abundant and widely distributed functional protein in mammals, which is important to wool growth (Hulmes, 2002; Izu et al., 2021). Fibrin and collagen are important to guide the formation and maintenance of hair fibers, and collagen protein family interacts to regulate the size of collagen fibers (Birk et al., 1990). In our research, most collagen mermbers were significantly enriched in ECM-receptor interaction pathway such as COL5A3, which displayed a increasing trend during wool growth. But other research have shown that COL5A3 was highly expressed in the sheep fetal skin, and its expression level was significantly reduced after birth (Nie et al., 2018). Therefore, we speculate that there are differences in COL5A3 expression between sheep and goat skin with growth. In our results, the fibronectin also showed a upward tendency in the wool straightening process. The collagen and fibronectin may play different roles in wool bending. Collagen alpha family proteins affect hair morphology by regulating the proliferation and differentiation of epithelial stem cells (Bonod-Bidaud et al., 2011), and fibronectin affects wool growth through fibroblasts (Dzamba and Peters, 1991; Zhao et al., 2021). In the present study, we found that collagen alpha family proteins and fibrin occupy an vital status, which may play a key role in wool bending of Zhongwei goat.

Due to complex post-transcriptional regulatory mechanisms in the organism, gene expression at transcript level and translational level are not always consistent (Ideker et al., 2001). To achieve the complementarity of data, we performed transcriptomic analysis on the same batch of samples. The integrative multi-omics analysis would be helpful for revealing the comprehensive molecular mechanism underling wool bending. The joint analysis of transcriptome and proteome data can provide more comprehesive gene expression information (McArdle and Menikou, 2021). Some previous studies have shown a moderate or low positive correlation between the transcriptome and the proteome (Park et al., 2019). We performed correlation analysis of overlapping DEGs, DEPs and PRM quantitative DEPs. In the three comparison groups, COL6A1, COL6A2, CRNN, TNC and LOC102178129 were found to be significantly differential expressed at the transcriptomic and proteomic level with the similar trends. Previous studies have indicated that the lack of collagen VI in mice delay hair circulation and growth under physiological conditions (Chen et al., 2015; Chen et al., 2016). The most abundant form of collagen VI is composed of three α1 (VI), α2 (VI) and α3 (VI) chains encoded by different genes (*COL6A1, COL6A2, COL6A3*), which may play a key role in the stem cell wall (Gara et al., 2008). CRNN is located primarily in the upper layers of differentiated squamous tissues and plays an important role in epidermal differentiation (Mlitz et al., 2014). Overexpression of *CRNN* can increase phosphorylation and activation of phosphoinositide 3-kinase and Akt (Li et al., 2019). *TNC* is leading-edge gene of the PI3K-Akt pathway in skin fibrogenesis (Moon et al., 2019; Barrera et al., 2020). The structural characteristics of wool fibrosis have a great influence on the wool bending (Harland et al., 2018). *LOC102178129*, as a novel identified gene, deserves further study in the future. Afterwards, we will further verify the effects of the differential proteins mentioned above in the ECM-receptor interaction and PI3K-Akt signaling pathway.

In order to clarify the function of the candidate genes, we selected *COL6A1* for functional verification at the cellular level based on previous studies and the function of the candidate genes. Hair follicles are skin accessory organs that regulate hair growth and have important roles in the formation of hair curved shapes. So DPCs was used to explore the potential function of *COL6A1* in hair follicles growth *in vitro.* The results demonstrated that the knockdown *COL6A1* inhibited *β-catenin* mRNA expression and VIMENTIN protein expression. *β-catenin* and VIMENTIN have been reported to play important roles in hair follicles. In addition, MTT assay aslo showed knockdown *COL6A1* can remarkablely affect DPCs proliferation. Therefore, we speculate that COL6A1 is involved in regulation of wool growth through promote the proliferation of DPCs.

## Materials and Methods

### Experimental Samples and Ethics Statement

All animal experimental procedures were approved by the Ministry of Agriculture of the People’s Republic of China and Institute of Animal Science, Chinese Academy of Agricultural Sciences and were performed according to the guidelines for the care and use of experimental animals established by this ministry. Before collecting samples, we obtained the permission of Ningxia Zhongwei Goat Conservation Farm. Three unrelated Zhongwei goats located at a Zhongwei goat breeding farm in Ningxia, China, were randomly selected. All the animals were raised in the same condition. The scapular skin at the age of 45 days (T1), 108 days (T2), and 365 days (T3) from three individuals were collected, and 2 cm^2^ of skin tissue was collected from the scapular region using a sterilized scalpel blade. The tissues were placed in a cryotube and stored in liquid nitrogen. Samples were immediately stored in RNAlater (Thermo Fisher Scientific, New York, United States) and kept at −80°C until further processing. All wounds were treated with Yunnan Baiyao Powder to stop bleeding (China Yunnan Baiyao Group Co., Ltd., Kunming, China). Ethical approval for animal survival was provided by the animal ethics committee of the Institute of Animal Science, Chinese Academy of Agricultural Sciences (IAS-CAAS) with the following reference number: IASCAAS-AE-03, on 1 September 2014.

### Extraction of Total Protein From Zhongwei Goat Skin Tissue

First, nine skin samples were ground into a powder in liquid nitrogen, and then the powder were added with lysis buffer (8 M urea, 1% protease inhibitor) and lysed by ultrasound. Next, cell debris were removed by centrifuge at 12,000 g for 10 min at 4°C, and the supernatant was transfered to a new centrifuge tube. Finally, the filtrate was collected and protein quantification was performed using BCA ProteinAssayKit (Abcan, China), and the processed samples were stored at−80°C.

### Pancreatic Enzymatic Hydrolysis and TMT Labeling

Thirty microliters of high-quality protein solution per sample was treated with pancreatic enzyme, and the filtrates was obtained, and then peptide quantification was carried out (measured by optical density at 280 nm; OD280). For each sample, 100 µg of peptide was labeled with TMT using the TMT Mass Tagging kits and reagents (Thermo Fisher Scientific, United States) according to the manufacturer’s instructions (Chen et al., 2017).

### High Performance Liquid Chromatography

The tryptic peptides were fractionated into fractions by high pH reverse-phase HPLC using Thermo Betasil C18 column (5 μm particles, 10 mm ID, 250 mm length). Briefly, peptides were first separated with a gradient of 8%–32% acetonitrile (pH 9.0) over 60 min into 60 fractions. Then, the peptides were combined into six fractions and dried by vacuum centrifuging.

### Liquid Chromatography-Mass Spectrometry Analysis

The tryptic peptides were dissolved in 0.1% formic acid (solvent A), directly loaded onto a home-made reversed-phase analytical column (15-cm length, 75 μm i.d.). The gradient was comprised of an increase from 6% to 23% solvent B (0.1% formic acid in 98% acetonitrile) over 26 min, 23%–35% in 8 min and climbing to 80% in 3 min then holding at 80% for the last 3 min, all at a constant flow rate of 400 nL/min on an EASY-nLC 1000 UPLC system.

The peptides were subjected to NSI source followed by tandem mass spectrometry (MS/MS) in Q ExactiveTM Plus (Thermo) coupled online to the UPLC. The electrospray voltage applied was 2.0 kV. The m/z scan range was 350–1800 for full scan, and intact peptides were detected in the Orbitrap at a resolution of 70,000. Peptides were then selected for MS/MS using NCE setting as 28 and the fragments were detected in the Orbitrap at a resolution of 17,500. A data-dependent procedure that alternated between one MS scan followed by 20 MS/MS scans with 15.0s dynamic exclusion. Automatic gain control (AGC) was set at 5E4. Fixed first mass was set as 100 m/z.

### Data Analysis and Identification of Differentially Expressed Proteins

The resulting MS/MS data were processed using Maxquant search engine (v.1.5.2.8). Retrieval parameter Settings: The database is *Capra hircus*_NCBI_1901 (42687 sequences), and the inverse library is added to calculate the false positive rate (FDR) caused by random matching. Trypsin/P was specified as cleavage enzyme allowing up to 4 missing cleavages. The mass tolerance for precursor ions was set as 20 ppm in First search and 5 ppm in Main search, and the mass tolerance for fragment ions was set as 0.02 Da. Carbamidomethyl on Cys was specified as fixed modification and acetylation modification and oxidation on Met were specified as variable modifications. FDR was adjusted to <1% and minimum score for modified peptides was set >40. Proteins with a |fold change|>1.3 and *p*-value<0.05 in three comparison groups (T2 VS. T1, T3 VS. T2, T3 VS. T1) were considered DEPs.

### Functional Annotation of Differentially Expressed Proteins

In order to explore the potential roles of DEPs in the three stages, we conducted the Gene Ontology GO and KEGG analysis for DEPs. Functional annotation of GO was performed using InterProScan (v.5.14–53.0 http://www.ebi.ac.uk/interpro/). For functional annotation of KEGG, the KAAS (v.2.0 http://www.genome.jp/kaas-bin/kaas_main) was used to map DEPs to the KEGG genes database, and then the mapped proteins were categorized based on KEGG. Finally, the pathways involving those classified proteins were obtained automatically. GO term or KEGG pathway with *p* < 0.05 after fisher’s exact test was considered significantly enriched.

Analysis of protein-protein interactions (PPIs) is an effective strategy for exploring proteins of unknown function. According to the result of KEGG Enrichment, the key DEPs were selected to draw the interactive network. Firstly, the differential proteins of goat skin samples in the three periods were screened. Protein interaction network were constructed using the STRING (v.10.5). The confidence score> 0.7 (high confidence) was set as the screening criterion. The R package “networkD3” tool was used to visualize the differential protein interaction network.

### PRM Validation for Differentially Expressed Proteins

To validate the accuracy of TMT-based proteomic results, the parallel reaction monitoring (PRM) method was applied to examine the protein abundances. The peptides were dissolved in liquid chromatography mobile phase A (0.1% formic acid solution) and separated using the EASY-nLC 1,000 ultra-high performance liquid system. Then, the samples were injected into the NSI ion source for ionization and the analysis by Q ExactiveTM Plus mass spectrometry. Finally, the resulting MS data were processed using Skyline (v.3.6) (MacLean et al., 2010).

### Extraction of Total RNA, RNA Sequencing and Data Analysis

The total RNA was extracted according to the instruction of RNeasy Plus Universal mini kit (Qiagen, Germany). The RNA concentration was evaluated using a NanoDrop spectrophotometer (NanoDrop Technologies, United States), and the RNA integrity was verified using Agilent 2,100 bioanalyzer (Agilent, United States). After RNA quality control, a total of 1.5 μg RNA per sample was used as input material for subsequent sequencing. RNA library construction and sequencing were performed using the Illumina Nova seq platform from Berry NGS (Beijing, China). The reads containing joints and low-quality reads were removed. Clean reads was compared to the reference genome (ftp://ftp.ncbi.nlm.nih.gov/genomes/refseq/vertebrate_mammalian/Capra_hircus/latest_assembly_versions/GCF_001704415.1_ARS1) by TopHat and Cufflink (Trapnell et al., 2012). For differential expressed genes (DEG), the screening criteria was set as *p*-value ≤0.05 and |fold change|> 1.5. The hierarchical clustering of DEG was performed using R software package ggplot2.

### Integrated Analysis of Transcriptome and Proteome

In order to achieve data complementarity and obtain more comprehensive gene expression information of Zhongwei goats during three skin development periods, the proteomic results, PRM protein quantitative results and transcriptomic results were jointly analyzed. Firstly, the name of the different protein was converted to a gene number through NCBI (https://www.ncbi.nlm.nih.gov/). Then, we combined the DEPs and DEGs. At last, we verified the overlaped DEGs by q-PCR.

### Q-PCR to Verify Candidate Genes

q-PCR was performed to verify the potential candidate genes. Based on the joint analysis of the transcriptome and proteome, we selected five differential genes for qPCR analysis. cDNA was synthesized using PrimeScript TM RT reagent kit with gDNA Eraser (Perfect Real Time) kit (Takara, Dalian, China). The qPCR were performed on ABI 7500 (Applied Biosystems, United States) using SYBR Premix Ex Taq Π (Tli RNaseH Plus) kit (Takara, Dalian, China) according to the manufacturer’s protocol. Thermal cycling consisted of an initial step at 95°C for 10 min followed by 40 cycles at 95°C for 30 s and 62°C for 30 s. The levels of candidate genes were determined relative to the expression levels of GAPDH. And q-PCR was performed using the following reaction system: 10 μl of SYBR Premix DimerEraser, 2 μl of cDNA, 0.4 μl Rox Reference Dye, 0.8 μl of upstream and downstream primers and 6 μl of ddH2O without RNase. The relative expression levels of the RNA were determined with the 2^–ΔΔCT^ method based on the cycle threshold (Ct) values. The primers were detailed in Supplementary Table S2.

### Cell Transfection

SiRNA were formulated in 20 µM stock solutions for transfection experiments according to siRNA product instructions (Shanghai Generay, Shanghai, China). The siRNA sequences were showed in Supplementary Table S3. To explore the function of candidate gene, siRNA and si-NC were transfected into Dermal papillary cells (iCell Bioscience Inc), when the revived cells grow up to about 90%, cells were seeded to 6-well plates. Transfection was performed when the cells grew up to about 80%. We transfect siRNA using Lipofectamine™ 3000 (Invitrogen).

### Cell Proliferation Assays

The cell viability upon transfection siRNA was assessed by MTT (Solarbio, Beijing). SiRNAs were seeded in 96-well plate and after 48 h assays, then checked following the manufacturer instructions. The viability of the cells was quantified as the percentage (%) of living cells relative to untreated cell (Bejaoui et al., 2021).

### Western Blot Analysis

Protein extraction and western blot were conducted as previously described (Hong et al., 2019). Briefly, total proteins from PFFs were homogenized using RIPA buffer. Protein concentrations were determined using the BCA Protein Assay Kit (Thermo Pierce, United States). Proteins were separated by 10% sodium dodecyl sulfate polyacrylamide gel electrophoresis, transferred to a polyvinylidene fluoride membrane (Millipore, United States), and then incubated with antibodies (Vimentin, proteintech; GAPDH, CST) overnight at 4°C and then with HRP-conjugated secondary antibody for 1.5 h at room temperature. Pictures were captured by an imaging system (UVP, United States).

## Conclusion

We provide the firstly comprehensive proteomic and transcriptomic data for Zhongwei goat skin with different wool bending phenotype at three development stages. A serious of potential mRNAs and proteins response to Zhongwei goat wool bending have been identified. These differential mRNAs and proteins in goat skin across three different stages were mainly enriched in signal pathways such as ECM-receptor interaction, PI3K-Akt signaling pathway, protein digestion and absorption. Correlation analysis of transcriptomics and proteomics showed COL6A1, COL6A2, CRNN, TNC and LOC102178129 may be involved in regulating wool bending of Zhongwei goat. Moreover, COL6A1 may effect wool growth or wool phenotype through regulation of hair follicle growth cycle. Our results provide a useful transcriptomic and proteomic resource and broad understanding of mechanism underlying animal wool bending.

## Data Availability

The original contributions presented in the study are publicly available. This data can be found here: https://ngdc.cncb.ac.cn/, accession number CRA005694 and OMIX ID: OMIX857.

## Abbreviations

DEP, differentially expressed proteins; DEG, differentially expressed genes; Seq sequence; GO, Gene Ontology; KEGG, Kyoto Encyclopedia of Genes and Genomes; PPI, Protein-protein interaction; RT-qPCR, Quantitative real time polymerase chain reaction; DPCs, dermal papilla cells.
