# Supplementary material for: Integration Analysis of Transcriptome and Proteome Reveal the Mechanisms of Goat Wool Bending
Source: Front Cell Dev Biol. 2022 Apr 1;10:836913. doi: 10.3389/fcell.2022.836913 (PMC9011194; doi:10.3389/fcell.2022.836913)
Supplement: Supplementary file 1 [file DataSheet1.pdf]

Supplementary

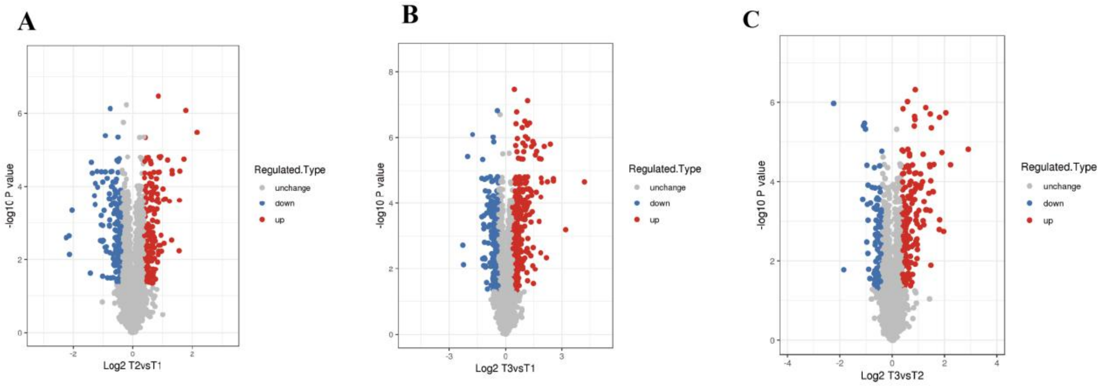

Figure S1 | Quantitative volcano diagram of differentially expressed proteins. The horizontal axis is the log-log converted value of the relative quantitative protein value, and the vertical axis is the log-plot converted value of the p-value value of significance test. Red dots indicate significantly differentially expressed up-regulated proteins, and blue dots indicate significantly differentially expressed down-regulated proteins. **(A)** T2 VS T1. **(B)** T3 VS T1. **(C)**T3 VS T2.

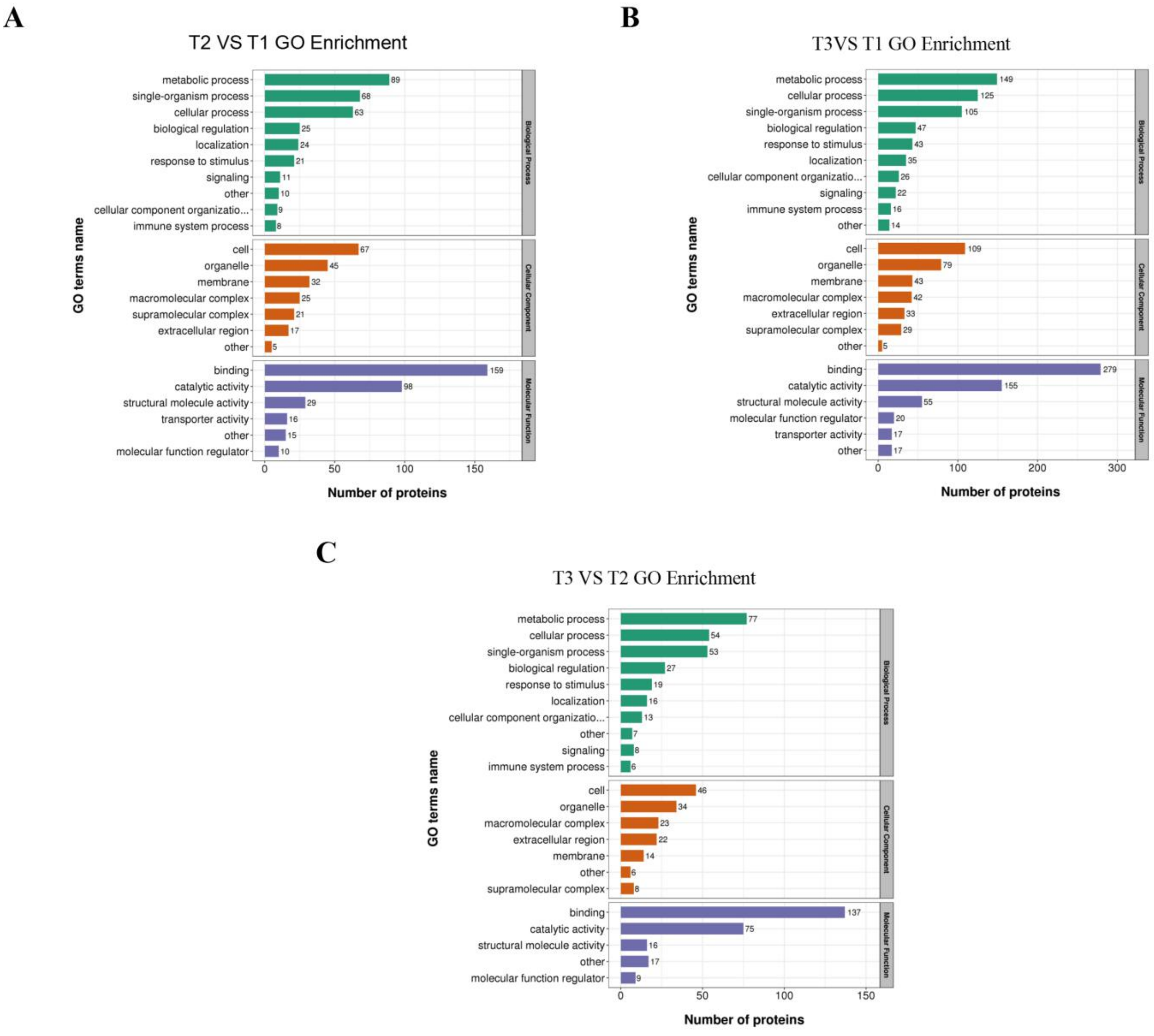

Figure S2 | The GO enrichment results. The abscissa is the number of DEPs, the right side of the ordinate is the

GO classification, and the left side is the GO term. **(A)** T2 VS T1. **(B)** T3 VS T1. **(C)** T3 VS T2.

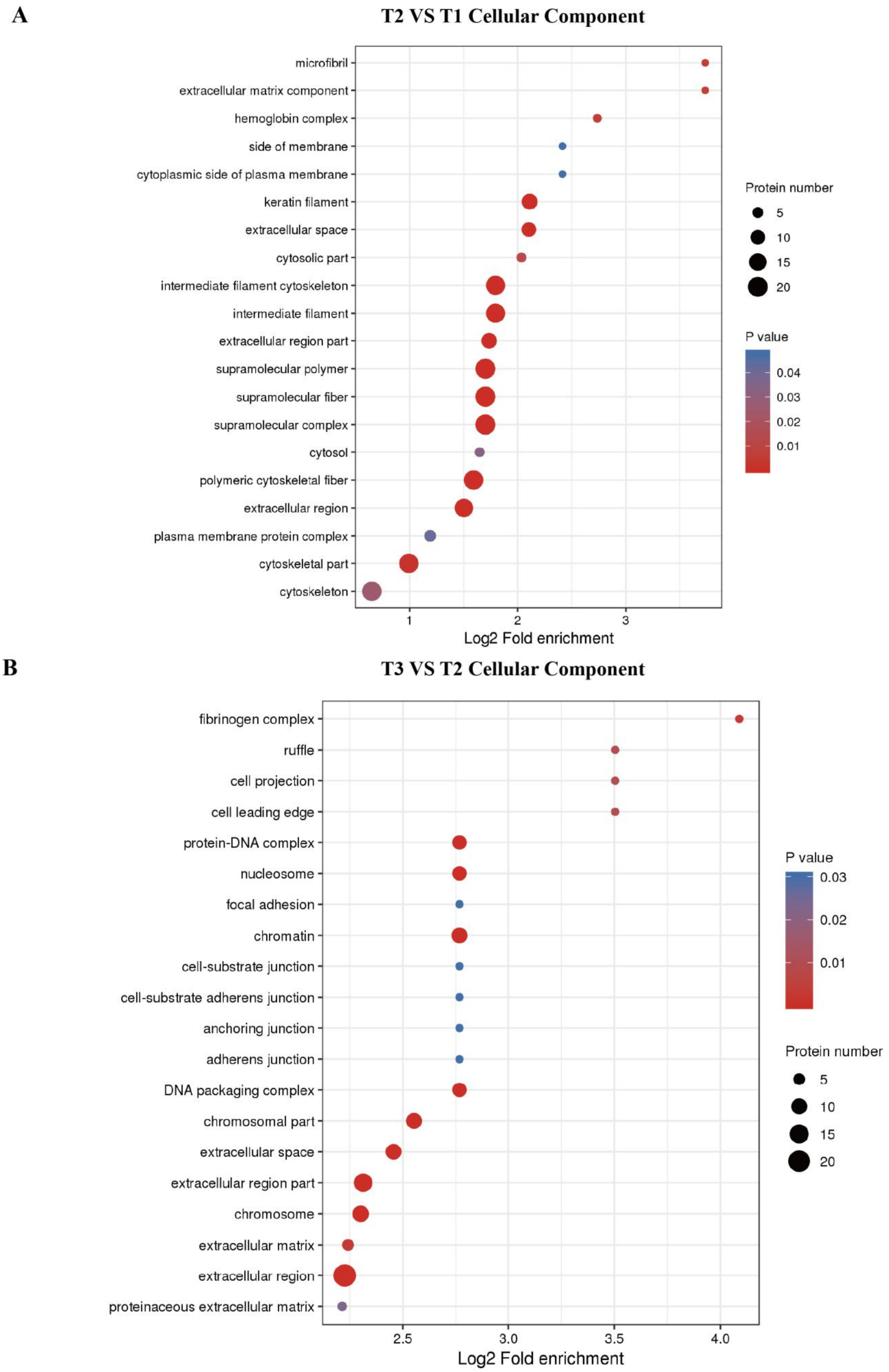

Figure S3 | GO terms enrichment of Cellular Component iterm. The size of the points represents the number of DEPs, color indicates enrichment and the redder the color, the more enrichment. **(A)** T2 VS T1 **(B)** T3 VS T2

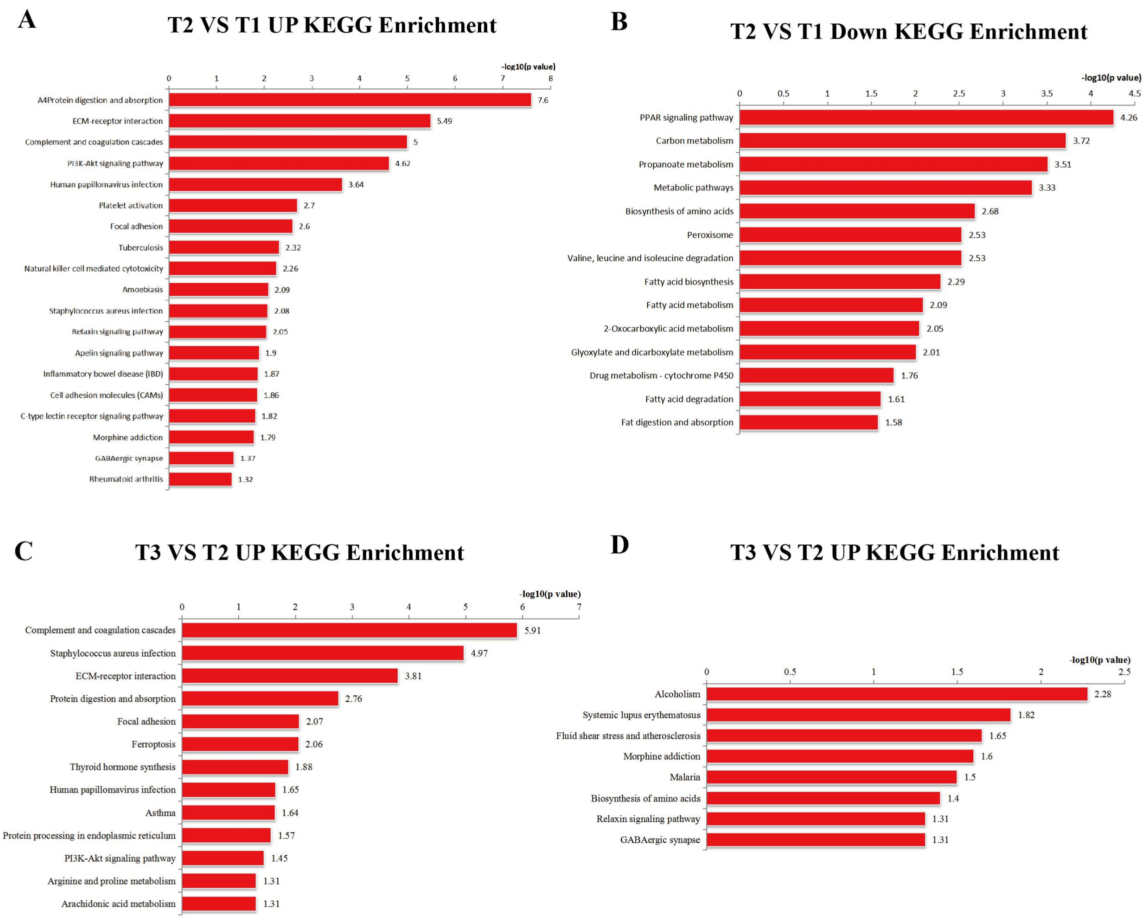

Figure S4 | The up/down-regulated DEPs KEGG Pathway enrichment. The abscissa is the enrichment of DEPs, the left side of the ordinate is the KEGG pathway. **(A)** T2 VS T1 up KEGG Enrichment **(B)** T2 VS T1 down KEGG Enrichment **(C)** T3 VS T2 up KEGG Enrichment **(D)** T3 VS T2 down KEGG Enrichment.

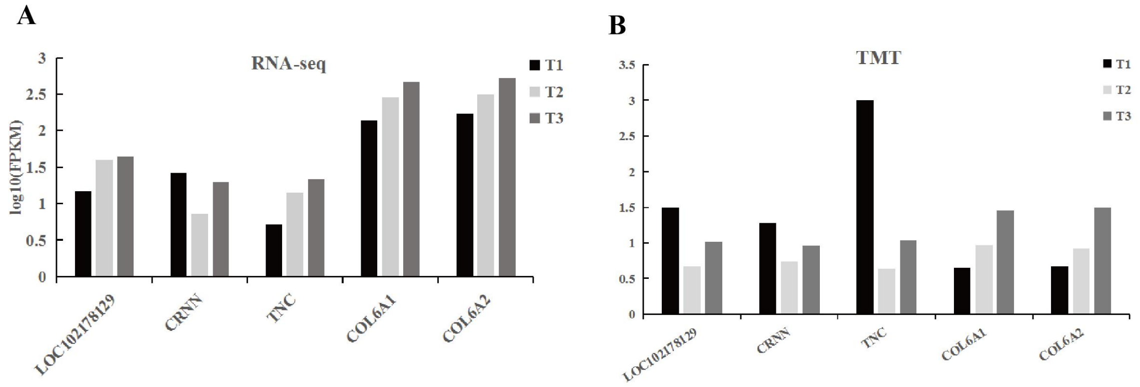

Figure S5 | Verification of differentially expressed mRNAs **(A)** and mRNAs **(B)** by proteins.

Table S1 Statistics of protein identification results

Total spectrum	Matched spectrum	Peptides	Unique peptides	Identified proteins	Quantifiable proteins
341563	59848 (17.5%)	33965	31553	4994	3798

Table S2 Fluorescent quantitative PCR primer sequences

Gene name	upstream Primer 5'→3'	downstream Primer 5'→3'
TNC	GATGCAGCCTACACGGTCAA	AGTCCAGGTCACGGTTGTTG
CRNN	TGGGTTGATGACCACACGAG	AGGAATACAGTCCCCTGGCT
LOC102178129	ATCCACCAGTATGTCCCCCA	CCATGCTCTTTAATGAGTGTCATC
COL6A2	AATCTCCAGCTTTCTGTCCTGG	TTAAGTTCCCTTCCTCCACAC
COL6A1	ATCGGGCCAAAAGGATACCG	TCTTCACCCCTCTCACCCAT
GAPDH	GCAAGTTCCACGGCACAG	GGTTCACGCCCATCACAA

Table S3. The siRNA sequences.

siRNA	Sense(5'-3')	Antisense(5'-3')
Si-COL6A1-1	GGUGCUUGUCAGUGACCUATT	UAGGUCACUGACAAGCACCTT
Si-COL6A1-2	GAACAAGUGUGUUGUUCUUTT	AAGAACAACACACUUGUUCTT
Si-COL6A1-3	AGAUCUGUAUAGAUAAGAATT	UUCUUAUCUAUACAGAUCUTT
